# Health behaviors and union dissolution among parents of young children: Differences by marital status

**DOI:** 10.1371/journal.pone.0182628

**Published:** 2017-08-10

**Authors:** Jess M. Meyer, Christine Percheski

**Affiliations:** Department of Sociology, Northwestern University, Evanston, IL United States of America; University of Alabama at Birmingham College of Arts and Sciences, UNITED STATES

## Abstract

Previous research finds that marriage is associated with better health and lower mortality, and one of the mechanisms underlying this association is health-related selection out of marriage. Using longitudinal survey data from 2,348 couples from the Fragile Families and Child Wellbeing Study, we examine whether certain health behaviors—smoking and binge drinking—are associated with risk of union dissolution among couples with young children. We use discrete time hazard models to test whether associations between health behaviors and union dissolution differ between married and cohabiting parents. We find no statistically significant association between binge drinking and union dissolution for either cohabiting or married couples. Parental smoking, however, is associated with union dissolution. On average, married and cohabiting couples in which both parents smoke have a higher risk of union dissolution than couples in which neither parent smokes. Additionally, father’s smoking (in couples in which the mother does not smoke) is associated with union dissolution, but only for married couples. These findings illustrate the importance of considering the health behaviors of both partners and provide further evidence of differences in union dissolution dynamics between married and cohabiting couples.

## Introduction

Married individuals are less likely to engage in certain risky behaviors with potentially negative health consequences than unmarried individuals [1]. Empirical evidence suggests that marriage causes individuals to change their health behaviors, including causing a reduction in risky health behaviors, such as binge drinking and illegal drug use (see reviews in [2,3]). Previous research also suggests that engaging in certain health behaviors, such as heavy drinking, is negatively associated with likelihood of entering marriage [4]. Health behaviors also impact marital status by affecting social selection out of marriage [5]. Being married may cause individuals to participate in fewer risky health behaviors, but individuals who engage in risky health behaviors are more likely to divorce than individuals who do not engage in these behaviors [5].

Risky health behaviors may be associated with union instability for a number of reasons. First, such health behaviors may impede an individual’s ability to enact normative family roles [6], and individuals who cannot fulfill normative roles may be perceived as less desirable partners. For example, frequent binge drinking may interfere with an individual’s ability to parent responsibly, placing children’s safety at risk and/or causing the co-parent to shoulder more of the caretaking responsibilities. The extent to which risky health behaviors interfere with family role expectations may differ by gender, in part because norms about parenting differ by gender. Many risky health behaviors are also more normative for men [7], and behaviors that are more common may be perceived as less incompatible with gender-specific family norms.

Second, risky health behaviors may incur substantial short-term and long-term financial costs or instability. In the short-term, some health behaviors may cause a partner to miss work or lose employment, and the behavior itself may be expensive, as in the case of a cigarette smoking habit or frequent bouts of heavy drinking. In the long-term, risky health behaviors may impair health and cause serious morbidity or disability, potentially decreasing employment earnings and incurring high health care costs. Financial strain, in turn, might negatively impact the quality of a couple’s relationship [8].

Third, risky health behaviors may impact the quality and tone of interactions between partners, perhaps especially in couples in which only one partner engages in the risky behavior. Because risky health behavior may interfere with family roles, incur financial costs, and negatively impact health, the partner who is not binge-drinking or smoking may try to exert social control over the partner who is engaging in the risky behavior (see [1,9] regarding spousal social control of health behavior). Attempts by one partner to monitor or sanction the other partner's behavior might lead to conflict and a deterioration of relationship quality (for example, [10] shows psychosocial effects of spousal diet control attempts).

Another possibility is that risky health behaviors are not themselves causally related to marital or union stability but rather are correlated with other characteristics that are associated with union dissolution. For example, cigarette smoking is more common among less educated adults in the United States, and education is an important predictor of marital stability in the United States [11]. Some risky health behaviors—like binge drinking—are also associated with particular health conditions, such as depressive symptoms [12], which previous research has found are predictive of marital dissolution [13]. Risky health behaviors could also affect health status, and it may be the resultant health conditions, not the risky behaviors per se, that impact union stability. For example, previous research finds that poor self-rated health is associated with union dissolution [14].

Psychosocial factors at the couple and individual levels might also produce non-causal associations between health behaviors and union dissolution. For instance, if poor relationship quality generates psychosocial stress, individuals in lower quality relationships might be more likely to smoke, as previous research has found that stress is linked to smoking [15]. At the individual level, personality characteristics such as neuroticism are associated with both smoking behavior and marital quality [16,17].

Studies that have empirically examined the relationship between risky health behaviors and marital dissolution have mixed findings. For example, in a sample pooling men and women together, Collins, Ellickson, and Klein [18] find that frequency of alcohol intoxication is positively associated with risk of divorce among young, married individuals in the U.S. In contrast, in a U.K. sample, Cheung [19] finds that harmful drinking is associated with increased likelihood of being divorced for women, but not for men. Additionally, Cheung [19] finds that women who were light smokers were significantly more likely than non-smokers to be divorced ten years later, but among men, heavy smokers were significantly less likely than non-smokers to be divorced. Fu and Goldman [5] also find a complicated pattern of results regarding risky health behaviors and divorce, in a U.S. sample (that excluded blacks and Hispanics). On the whole, their findings suggest that smoking is positively associated with risk of divorce for women and men, and that for men married longer than four years, moderate drinking is negatively associated with divorce risk. Finally, another study finds that a “mismatch” in alcohol use is associated with an increased likelihood of marital dissolution [20].

Among studies analyzing the association between health behaviors and future dissolution risk, relatively few examine couples with children specifically. One exception [6] shows evidence supportive of role incompatibility between parenthood and marijuana use, but does not find statistically significant interactions between parental status and marijuana use in predicting union dissolution. A more recent study by Tach and Edin (2013) [21] finds that among couples with children, problematic drinking or drug use (i.e., that which interferes with work or relationships) by mothers (but not fathers) is associated with higher likelihood of future union dissolution, but only among married couples.

The prevalence of cohabiting relationships has risen dramatically over the past four decades [22], and almost one-quarter of children are now born to unmarried cohabiting couples [23]. Given the importance of family stability to child wellbeing (e.g. [24,25]), it is particularly important to understand whether risky health behaviors are associated with union stability among parents. Relatively little research has examined whether the associations between risky health behaviors and union stability are similar for married and cohabiting couples, and to our knowledge, no study has explicitly analyzed how the associations of specific health behaviors with union dissolution differ between married and cohabiting couples with young children.

Although marriage and cohabitation are both sexual co-residential relationships, there are still notable differences between the two union types in the United States. These include differences in the legal rights and obligations of the partners, social and financial support from kin [26,27], and norms about housework [28], pooling money [29], and parenting [30,31]. Additionally, cohabiting parents separate at a much higher rate than married parents [21]. These differences may alter the associations between risky health behaviors and union stability.

In this article, we examine how health behaviors are associated with union dissolution for married and cohabiting couples with children. Using nine years of data from the Fragile Families and Child Wellbeing Study, a study of parents with children born in 1999 and 2000 who live in urban areas in the United States, we examine the association between union stability and two health behaviors: smoking and binge drinking. In contrast to earlier research on this topic using data from the Fragile Families and Child Wellbeing Study [21,32], we examine specific measures of cigarette use and binge drinking among mothers and fathers. We investigate three questions: 1) Are risky health behaviors (smoking and binge drinking) associated with (future) union dissolution? 2) Do the associations of these health behaviors with union dissolution differ between married and cohabiting couples? 3) Do the associations between these health behaviors and union dissolution differ by the gender of the partner engaging in the behavior?

On the one hand, given the stronger norms for men’s and women’s behavior within the institution of marriage, we might expect risky health behaviors to be more predictive of union dissolution among married couples. Tach and Edin’s (2013) [21] research, for example, suggests that mothers’ drinking or drug problems are predictive of union dissolution among married couples, but not unmarried couples (in their analysis, this category included both cohabiting and dating couples). On the other hand, cohabiting unions are more likely to dissolve than marriages, so to the extent that a risky health behavior produces strain within couples, we might expect this behavior to be more predictive of dissolution among cohabiting unions.

Our expectations regarding gender differences in the associations between risky health behaviors and union dissolution are more one-sided. Given gendered norms of parenting behavior, and the incompatibility of risky health behaviors with supervising children, we expect that mothers’ risky health behaviors will be more predictive of union dissolution than similar behavior by fathers.

We do not hold a priori expectations regarding whether binge drinking or smoking will be more predictive of union dissolution. Both activities can incur a financial cost, which varies according to frequency of engaging in the health behavior. Binge drinking could more severely limit a parent’s ability to supervise or transport children to school, activities, etc. Smoking, however, could impact children’s health via second-hand smoke exposure [33], potentially interfering with expectations about good parenting.

Thus, the overall aim of this research is to analyze whether associations between health behaviors and union dissolution differ by marital status, but we also explore differences by gender and the specific health behavior examined. We achieve this objective by using discrete time hazard models to examine risk of union dissolution among a sample of parents with young children in U.S. urban areas. Our findings demonstrate the importance of considering marital status, gender, and disaggregated health behavior measures in research connecting health behavior to union dissolution.

## Data and methods

### Data

This analysis was deemed exempt by the IRB at the authors’ university as it uses publicly available data. Our data come from the Fragile Families and Child Wellbeing Study, a birth cohort study that includes around 5,000 children born in twenty large U.S. cities in 1999 and 2000. The Fragile Families sample contains a larger number of cohabiting couples than is available in many other datasets, as the study oversampled unmarried births. More details about the sampling methodology are available in [34]. Readers should note that although these data are not representative of the entire U.S. population, they are potentially informative regarding a substantial segment of it; in the year 2000, 22% of all U.S. births and 29% of non-marital births were in urban areas [35].

Another exceptional feature of the Fragile Families study is that both mothers and fathers were interviewed, even if the parents were unmarried. (There are no mothers who report a same-sex partner at the baseline wave of the study.) Thus, these data include a much larger sample of cohabiting couples than most datasets with health information for both partners. The baseline interviews were conducted shortly after the child’s birth (usually while the mother and infant were still in the hospital), and subsequent interviews were conducted 1 year, 3 years, 5 years, and 9 years later.

Because the same health behavior questions were not asked of both parents at the baseline interview, we define the start of our observation window as the 1-year interview. Additionally, the mothers’ baseline survey asked about health behaviors during pregnancy, and associations between health behaviors during pregnancy (when mothers’ health behaviors may impact the fetus) and union dissolution might differ from the associations at other points in the life course. Because the start of our observation period is one year after the baseline interview, couples whose unions dissolved by their child’s first birthday are not included in our main sample. Of the 4,898 families who participated in the Fragile Families and Child Wellbeing study, 2,997 included couples in a residential union at the 1-year interview or, if they did not participate in the 1-year interview, in a residential union at baseline. Of these families, our analytic sample includes couples in which both partners participated in the 1-year interview (n = 2,348). We use multiple imputation techniques (with twenty iterations) to address missing data on predictor and covariate variables. Of our sample, 88.37% of couples had one or no predictor or control variables with missing data. Most variables included in our analysis had relatively few missing cases. Of the health behavior variables, all variables had 5 or fewer cases with missing values.

#### Variables

We measure marital status using a dichotomous indicator of whether the couple is married or in an unmarried, cohabiting union at the 1-year interview. Our outcome measure is an indicator that the couple has separated between survey waves (i.e. between the 1 year and 3 year survey, between the 3 year and 5 year survey, or between the 5 and 9 year survey). We use mothers’ reports when available and supplement with fathers’ reports if the mother did not participate in the interview wave.

We examine two health behaviors, each measured for both members of the couple at the 1-year interview when the child was approximately one year old. These behaviors are 1) whether the respondent reported smoking cigarettes in the previous month (a dichotomous variable); and 2) whether the respondent reports one or more days in the previous month on which they drank five or more alcoholic beverages (a dichotomous variable). We distinguish between indicators of mothers’ and fathers’ health behaviors. Unfortunately, questions about binge drinking in later survey years are not directly comparable to the 1-year measure. Smoking measures are more comparable across waves, but, because of a survey implementation error, are not available at the 3-year interview for most parents. Given this limitation and the fact that less than 15% of mothers and fathers change smoking behavior between the 1-year and 5-year interviews, we use only the 1-year interview measures in our analysis.

For smoking, we construct a four-category smoking variable denoting the combination of paternal and maternal smoking behavior at the 1-year interview. The categories include: neither parent smokes, mother smokes only, father smokes only, and both parents smoke. (Maternal binge drinking is too rare to include the parallel measure for parental binge drinking in the analysis.)

The full set of control variables includes variables previously found to be associated with union dissolution (e.g. [11,21]). Mother’s characteristics include age (measured using five categories), race/ethnicity (with variables indicating whether she is non-Hispanic white, non-Hispanic black, Hispanic, or other), mother's education (with variables indicating whether she has less than a high school degree, high school, some college, or a college degree or more), her childhood family structure (with a variable indicating whether she lived with both parents at age 15), and whether she was previously married (an indicator variable). Father’s characteristics include measures of his involvement with the criminal justice system (an indicator of whether he has a criminal record), his current or previous military enlistment (a variable indicating military service), recent unemployment (measured with an indicator of whether he was not working for three or more weeks in the year before the birth), and whether he was previously married (an indicator variable).

Couple-level characteristics include: the age difference between the mother and father; whether the mother and father are of the same race/ethnicity (a variable indicating if they are homogamous); whether either or both parents are immigrants (an indicator variable); whether the mother has children with a previous partner (an indicator variable); whether the father has children with a previous partner (an indicator variable); whether they have other shared children together (an indicator variable); whether the couple considered abortion (coded yes if the mother says that she considered it or the father suggested it); and whether the mother reports that there was domestic violence in the relationship prior to the child’s birth (an indicator variable).

Controls for additional family characteristics include the following, operationalized as dichotomous variables: whether the couple’s infant has a serious health problem or disability, whether the couple owns their home, whether their income in the year between their child’s birth and the 1-year interview was below the federal poverty level, and whether both members of the couple attend religious services weekly. Additionally, we include two variables for union duration at the time of the child’s birth: a variable indicating that the couple had lived together one year or less, and an indicator variable for whether they had lived together for five or more years (with the reference category being couples who had lived together more than one year but less than five years at the time of the birth).

### Analysis

We use discrete time hazard models to analyze the association between health behaviors (as reported at the 1-year interview) and risk of union dissolution. The event outcome we model is union dissolution, defined as a married or cohabiting couple ceasing to live together. We assess this outcome at three points in time: the 3-year interview, the 5-year interview, and 9-year interview. Our unit of analysis is the couple, and each couple contributes an observation to our sample for each period at which they are at risk of separating, leading to a total of 5,811 observations. Couples in which a partner dies are treated as censored at the first wave that the death is reported, and are not included in the analysis at later waves. For couples in which neither partner completed an interview at a particular wave, we treat relationship status as censored for that particular wave and do not follow the couple at later waves.

Our modeling strategy is similar to that of Tach and Edin [21], and our first model, Model 1, can be represented using the following equation:

$$\log\left[\frac{P_{it}}{1-P_{it}}\right]=\beta_{1}{\left(Period\right)}_{it}+\beta_{2}{\left(Married\right)}_{i}{+\beta }_{3}{\left(Married\;X\;Period\right)}_{it}+\beta_{4}{\left(Smoking\right)}_{i}+\beta_{5}{\left(Drinking\right)}_{i}+\varepsilon_{it}$$

In this model, *P*_*it*_ represents the probability of union dissolution for couple *i* during discrete time period *t*, conditional on the couple being at risk of union dissolution during this period [21]. In this model specification, *Period* is a categorical variable that indicates to which of the three discrete measurement periods (between the 1-year and 3-year surveys, between the 3-year and 5-year surveys, or between the 5-year and 9-year surveys) couple *i* belongs at wave *t*. We do not include an intercept term in these regression models, so no one time period is treated as the reference period. Rather, the coefficient for each period represents the log odds of dissolution during that period when controlling for the other variables in the model. Marital status, parental smoking, and parental binge drinking do not vary within couples over time, as they are all measured at the 1-year interview. The *β*_2_ coefficient represents the difference in log odds of dissolution between married and cohabiting couples for the period between the 1-year and 3-year surveys. *β*_3_ captures interactions between marital status and period, demonstrating how differences in log odds of dissolution between married and cohabiting couples change over time. Associations of parental smoking variables with log odds of dissolution are captured by *β*_4_, and associations of parental binge drinking variables with dissolution are represented by *β*_5_.

In the second model specification (Model 2), we add interactions between the health behavior variables and marital status. In Model 3, we add the full set of control characteristics. (We checked for evidence of multi-collinearity among the control variables; we find no indication of such a problem.)

To aid interpretation of the substantive meaning of key coefficients, we calculate predicted probabilities of union dissolution. These probabilities are calculated for a couple with the reference values for the control characteristics (e.g., non-Hispanic white mother with a high school degree), except for the following: the mother lived with both parents in childhood, the parents are of the same race and ethnicity, and the age difference between parents is two years.

## Results

There are substantial differences in the demographic characteristics and resources of cohabiting and married couples, as well as in their risk factors for separation (see S1 Table). Table 1 shows rates of separation by relationship type. More than half of cohabiting couples split up during the observation window compared with approximately one-quarter of married couples. This higher rate of union dissolution among cohabiting couples is not unique to our sample; national data also show short union durations for cohabitors [36].

**Table 1:** Distribution of predictor and outcome variables by relationship type.

			Relationship type				Significant difference?
			Cohabiting		Married		
			(n = 1182)		(n = 1166)		
			Mean	standard error	Mean	standard error	
**Outcome**							
	*Couple splits by end of observation period*		.59	.01	.24	.01	*
**Health behavior predictor variables**							
	*Currently smokes cigarettes*						
		Mother	.30	.01	.15	.01	*
		Father	.48	.01	.25	.01	*
	*Binge drinking episode in previous month*						
		Mother	.07	.01	.04	.01	*
		Father	.28	.01	.25	.01	

*Source*: Fragile Families and Child Wellbeing Study

*Note*: Differences between married and cohabiting couples that are statistically significant at p < .05 are denoted by *.

Notably, health behaviors differ by gender and relationship type. Almost one-third of cohabiting mothers and one-half of cohabiting fathers reported smoking compared to approximately 15% of married mothers and one-quarter of married fathers. Binge drinking is rare among mothers, but is more common among fathers.

The results in Table 2 show how binge drinking and smoking associate with union dissolution. In Model 1, couples in which only the mother smokes, only the father smokes, or both parents smoke have higher odds of union dissolution, on average, than couples in which neither parent smokes. With regard to binge drinking, Model 1 shows no association between maternal or paternal binge drinking and union dissolution.

**Table 2:** Results from discrete time hazard models predicting union dissolution by parental health behaviors (OR = odds ratio, *β* = coefficient, s.e. = standard error).

	Model 1				Model 2			
	OR	*β*	s.e.	P > |t|	OR	*β*	s.e.	P > |t|
***Combined Parental Smoking*** *(Reference = Neither Parent Smokes)*								
Mother Smokes Only	1.66	.51	.14	***	1.52	.42	.17	*
Father Smokes Only	1.62	.48	.10	***	1.36	.30	.11	**
Mother and Father Both Smoke	1.90	.64	.10	***	1.63	.49	.12	***
Married X Mother Smokes Only					1.20	.18	.32	
Married X Father Smokes Only					1.71	.54	.20	**
Married X Mother and Father Both Smoke					1.70	.53	.23	*
***Binge Drinking***								
Mother Binge Drinks	.91	-.10	.16		.87	-.14	.18	
Father Binge Drinks	.98	-.02	.09		.92	-.08	.10	
Married X Mother Binge Drinks					1.16	.15	.37	
Married X Father Binge Drinks					1.19	.17	.18	
**Married**	.21	-1.58	.14	***	.15	-1.88	.16	***
***Discrete time periods***								
Between 1 and 3-Year Survey	.27	-1.30	.08	***	.30	-1.19	.09	***
Between 3 and 5-Year Survey	.23	-1.47	.10	***	.26	-1.36	.10	***
Between 5 and 9-Year Survey	.29	-1.24	.11	***	.32	-1.13	.11	***
Married X Between 3 and 5 Years	1.84	.61	.19	**	1.87	.63	.19	**
Married X Between 5 and 9 Years	1.61	.48	.20	*	1.66	.51	.20	*

*Source*: Fragile Families and Child Wellbeing Study (n = 2348)

*Note*: Statistical significance levels are denoted as follows:

***p < .001

**p < .01

*p < .05

When we add interactions between parental health behavior variables and marital status in Model 2, the coefficients for maternal smoking (only), paternal smoking (only), and both parents smoking remain statistically significant. Interactions of marital status with paternal smoking (only) and both parents smoking are statistically significant. We do not find statistically significant interactions between marital status and paternal or maternal binge drinking.

In Table 3, we show the results for Model 3, which includes the full set of control covariates. As in Models 1 and 2, Model 3 shows no associations between binge drinking and union dissolution for either cohabiting or married parents. With regard to smoking, the main coefficients for paternal and maternal smoking lose statistical significance. However, paternal smoking continues to interact with marital status (OR = 1.64, p < .05). The predicted probability of union dissolution between the 1 and 3 year interviews for married couples with the reference characteristics is .10 for couples in which the father (only) smokes compared with .05 for couples in which neither parent smokes. In Model 3, the main coefficient for both parents smoking maintains statistical significance (OR = 1.38, p < .05), but notably, the interaction of both parents smoking with marital status is not statistically significant. This shows that both parents smoking is equally predictive of union dissolution for married and cohabiting couples. For a cohabiting reference couple in which neither parent smokes, the probability of union dissolution between the 1-year and 3-year interviews is .16; for a couple with the same characteristics, this probability increases to .20 if both parents smoke.

**Table 3:** Results from discrete time hazard models predicting union dissolution by parental health behaviors, with covariate controls (OR = odds ratio, *β* = coefficient, s.e. = standard error).

	Model 3			
	OR	*β*	s.e.	P > |t|
***Combined Parental Smoking*** *(Reference = Neither Parent Smokes)*				
Mother Smokes Only	1.16	.15	.19	
Father Smokes Only	1.12	.11	.12	
Mother and Father Both Smoke	1.38	.32	.13	*
Married X Mother Smokes Only	1.32	.28	.33	
Married X Father Smokes Only	1.64	.50	.21	*
Married X Mother and Father Both Smoke	1.23	.21	.24	
***Binge Drinking***				
Mother Binge Drinks	.94	-.06	.20	
Father Binge Drinks	1.07	.07	.11	
Married X Mother Binge Drinks	1.02	.02	.39	
Married X Father Binge Drinks	1.13	.12	.19	
**Married**	.30	-1.21	.18	***
***Discrete time periods***				
Between 1 and 3-Year Survey	.31	-1.17	.21	***
Between 3 and 5-Year Survey	.28	-1.27	.22	***
Between 5 and 9-Year Survey	.38	-.98	.22	***
Married X Between 3 and 5 Years	1.86	.62	.20	**
Married X Between 5 and 9 Years	1.65	.50	.21	*
***Mother's characteristics***				
*Age (ref*: *24–27)*				
less than 20	1.51	.41	.14	**
20–23	1.17	.16	.11	
28–34	.74	-.30	.13	*
35 and older	.56	-.58	.18	**
*Race/ethnicity (ref*: *non-Hispanic white)*				
Black	1.58	.46	.11	***
Hispanic	.87	-.14	.13	
Other race	1.02	.02	.23	
*Education (ref*: *high school)*				
Less than high school	.91	-.10	.10	
Some college	.89	-.12	.11	
College or more	.58	-.54	.19	**
Lived with 2 parents during childhood	.87	-.14	.08	~
Previously married	1.02	.02	.16	
***Father's characteristics***				
Has criminal record	1.06	.06	.09	
Military service	1.15	.14	.13	
Unemployment spell	1.10	.10	.08	
Previously married	1.08	.08	.15	
***Couple-level Characteristics***				
Age difference between partners	.97	-.03	.01	*
Couple is the same race/ethnicity	.71	-.34	.12	**
Either parent is an immigrant	.65	-.43	.13	***
Mother has child with other man	1.01	.01	.09	
Father has child with other woman	1.23	.21	.10	*
Couple has other bio. children together	.91	-.09	.09	
Either considered abortion	1.12	.12	.09	
Domestic violence	1.41	.35	.21	~
Child has disability	1.86	.62	.28	*
Home ownership	.87	-.13	.11	
Below poverty line	1.16	.15	.09	~
Attend religious services weekly	.94	-.07	.12	
Couple has lived together less than 1 year	1.13	.12	.09	
Couple has lived together 5+ years	.92	-.08	.13	

*Source*: Fragile Families and Child Wellbeing Study (n = 2348)

*Note*: Statistical significance levels are denoted as follows:

***p < .001

**p < .01

*p < .05

~p < .10

### Supplementary analyses and robustness checks

Relationship quality may mediate the associations between health behaviors and union dissolution. To investigate this, we check whether the inclusion of a variable for poor relationship quality at the child’s birth (based on mother’s responses to items that comprise a supportiveness scale) alters our findings about the associations between health behaviors and union dissolution; it does not.

Given that maternal health behaviors during pregnancy may impact fetal health, we investigate whether having a child with a health problem or disability is a mechanism through which maternal health behaviors impact union stability. We check whether excluding the variable indicating a child health problem from our models modifies our main findings; it does not. Moreover, excluding couples who have a child with a health problem from the analysis also does not change our main findings.

In alternative model specifications, we include measures of each parent’s self-rated health and whether the parent exhibits high levels of (untreated) depressive symptoms using the shortened form of the CES-D checklist. Inclusion of these variables does not meaningfully change the associations between health behaviors and union dissolution. This suggests that the statistically significant associations we find between smoking and union dissolution are not spurious relationships caused by associations of self-rated health or depression with either the main predictors (smoking and binge drinking) or outcome variable (union dissolution). Interestingly, these results also suggest that self-rated health and depressive symptoms do not mediate the associations we find between smoking and union dissolution.

The beginning of our observation period is the 1-year interview, and some unions dissolved between the child’s birth and the 1-year interviews. To explore the selectivity of our sample, we examine the distribution of baseline health behaviors among all couples married or cohabiting at the child’s birth, disaggregated by whether the couple is included in our sample. The health behavior measures available at the baseline interview are not directly comparable to the measures we use in our main analysis, but provide information about possible selection. The results of this analysis are presented in S2 Table, and they provide some evidence of selection into our analytic sample on maternal and paternal smoking behavior. Among married couples, those in our sample are less likely to include mothers that smoked during pregnancy or fathers that smoked in the three months prior to the baseline interview. Given that couples in which mothers or fathers smoked are more likely to select out of our sample, it is possible that our main findings underestimate the association of smoking behaviors with union dissolution.

In an additional supplemental analysis, we run separate models for married and cohabiting couples; we do this because of evidence that the predictors of union dissolution are different for cohabiting and marital unions [21,37]. Our conclusions from these analyses parallel those from the models that include marital status interactions with health behaviors.

We also check whether our results are robust to using the couple’s relationship status at the child’s birth instead of at the start of our observation period (the 1-year interview). Consistent with our main analyses, findings from the Model 3 specification of this set of analyses show that couples in which both parents smoke and married couples in which the father (only) smokes have increased odds of union dissolution. In contrast to our main analyses (which use marital status at 1 year), this analysis also shows a statistically significant interaction term between maternal smoking and marital status (OR = 2.00, p < .05). This suggests that married couples in which the mother smokes have increased odds of union dissolution, but the same association does not hold for cohabiting mothers who smoke.

Finally, we check whether associations between health behaviors and union dissolution differ between couples cohabiting at the 1-year interview that later marry (*n* = 327) and cohabiting couples that do not marry during our observation period. Among couples that are cohabiting at the 1-year interview, we do not find statistically significant interactions of marrying in subsequent years with maternal smoking, paternal smoking, or paternal binge drinking. We may be underpowered to detect such interactions if they are small in magnitude. (Because of the small number of mothers who binge drink, we are unable to explore interactions between marrying and maternal binge drinking.)

## Discussion

Risky health behaviors might negatively affect individuals’ well-being and decrease the well-being of their partners and their children. In this paper, we investigate whether smoking or binge drinking among urban couples with a young child are associated with higher risk of future union dissolution. We expected that these health behaviors would be associated with an increased risk of union dissolution, as they might interfere with an individual’s ability to be a good partner or parent, and might incur substantial financial costs. We also posited that a "mismatch" between partners in risky health behaviors might cause conflict or strain that would lead to increased risk of dissolution. Previous research and theory suggest that the associations of these health behaviors with union dissolution might vary by the marital status of the couple and the gender of the partner engaging in the behavior.

In our analysis, we find that couples in which both parents smoke have an increased risk of union dissolution, and this increased risk does not differ by marital status. This finding is inconsistent with what we would expect if the “mismatch” hypothesis fully held. Additionally, this finding demonstrates the importance of considering dyadic pairings of health behaviors when predicting union dissolution, as opposed to examining each partner’s health behaviors in isolation from the other partner.

We also find evidence suggestive of gender differences in the relationships between smoking, marital status, and union dissolution. Notably, we do not find evidence in support of our expectation regarding the direction of gender differences; maternal smoking was not more predictive of union dissolution than paternal smoking. Instead, we find that married couples in which only the father smokes have an increased risk of union dissolution; however, the association between paternal smoking and dissolution risk does not hold for cohabiting couples. The fact that a “mismatch” in parental smoking behavior is predictive of dissolution only among married couples and only when the smoking parent is the father suggests that the mechanism linking paternal smoking to union dissolution might not be a “mismatch” per se, but rather gendered family dynamics surrounding health behaviors.

One potential explanation for our finding that married fathers' smoking is particularly predictive of union dissolution relates to partner social control of health behaviors. Prior research suggests that for men, being married is associated with a higher frequency of receiving health behavior social control from others [9]. Married men are also more likely than married women to report their partner (or ex-partner) as the person that most frequently performs such social control in their life [9]. If attempts at socially controlling a partner’s risky health behaviors are associated with relationship discord and union instability, our pattern of findings would be consistent with the gendered patterns of social control documented by previous research. Future research might examine whether cohabiting and married couples differ in gendered efforts to socially control health behavior, whether these efforts are more likely among couples in which only one partner engages in a particular risky health behavior, and how such efforts impact relationship quality.

We find no evidence that binge drinking is associated with union dissolution for either married or cohabiting parents. However, given the small number of mothers who binge drink in our sample, it is possible that our analysis is underpowered to detect associations among this group.

In further analyses, we explored possible mechanisms linking health behaviors to union dissolution, including relationship quality, self-rated health, and depression. We did not find any evidence that these account for the associations between smoking and union dissolution. Future research should investigate whether the association of smoking with union dissolution is spurious and generated by correlation with factors not captured by the Fragile Families Study, such as measures of personality dimensions or stress, that are associated with marital quality [15–17,38].

Our analysis focuses on parents who live in urban areas. Parents in U.S. cities are more racially diverse than parents not residing in cities. Cohabiting and married couples in urban areas may also differ from their less urban or rural counterparts on non-demographic characteristics. Thus, findings based on this sample may not generalize to the population of all parents in the U.S. This limitation is compensated for by the availability of health behavior information from both partners in the Fragile Families data, which is not typical for studies in this field [39]. Additionally, these data contain a larger number of cohabiting couples (over 1,000) than what most nationally representative datasets include.

In conclusion, our findings make two important contributions to the existing scholarly literature. First, consistent with previous research, we find that certain risky health behaviors are associated with romantic union dissolution. Like previous research, we find a somewhat complicated pattern of associations, with differences by the specific behavior examined (i.e., smoking versus binge drinking), by the gender of the partner engaging in the behavior, and by the marital status of the couple. Future research might fruitfully examine the precise mechanisms explaining why these associations differ across population subgroups. Second, our finding of differences in the association between smoking and dissolution by marital status adds to the growing body of research continuing to find differences in union dynamics between married and cohabiting couples, suggesting that marriage and cohabitation are still distinct family forms in the United States.

## Supporting information

S1 Table Descriptive statistics for characteristics of mothers, fathers, and couples by relationship type. *Source*: Fragile Families and Child Wellbeing Study (n = 2348). *Note*: Differences between married and cohabiting couples that are statistically significant at p < .05 are denoted by *.
(XLSX)

S2 Table Distribution of parents' health behavior variables at child's birth by baseline marital status and sample status. *Source*: Fragile Families and Child Wellbeing Study. *Note*: Statistically significant (p < .05) differences between sample and non-sample couples within marital status are denoted by *. Married and cohabiting couples at the child's birth are not included in our sample if they separate before the 1-year survey or if either parent did not complete a 1-year interview.
(XLSX)
